# An unexpected supraclavicular swelling

**DOI:** 10.1186/1477-7819-5-90

**Published:** 2007-08-04

**Authors:** Nishith NB Patel, Parin R Shah, Eliie Wilson, Puthcode N Haray

**Affiliations:** 1 Department of General Surgery, Prince Charles Hospital, Merthyr Tydfil. CF47 9DT, UK

## Abstract

**Background:**

Colorectal cancer is the third commonest cause of cancer death in UK. It commonly metastasises to the liver but rarely to small bones.

**Case presentation:**

We describe a case of a patient with adenocarcinoma of the descending colon who presented preoperatively with a right supraclavicular swelling. Subsequent imaging and cytology of the lesion revealed this to be a metastasis to the right clavicle resulting in a pathological fracture.

**Conclusion:**

This report describes the rare occurrence of a colorectal metastasis to the clavicle. It emphasises that although bone metastases from primary colorectal tumours are rare events, they tend to metastasise to small, non-weight bearing bones. It also discusses the utility of isotope bone scanning and that on certain occasions this imaging method may prove to be equivocal. In such circumstances, biopsy or magnetic resonance imaging is more sensitive for the detection of bone metastases.

## Background

Colorectal cancer is the third commonest cause of cancer death in the UK. It commonly metastasises to the liver (nearly 50%) [1], the other sites being lung, brain and bones. Metastasis to the clavicle is extremely rare and in this article, we report one such case of an unusual presentation of clavicular metastasis from a primary colonic malignancy not detected on the isotope bone scan.

## Case presentation

A 68 year old man presented with a change in bowel habit and weight loss. General and abdominal examinations were normal. A barium enema revealed a tight stricture in the descending colon, with no evidence of extra-colonic metastases on a staging CT scan. A multi-disciplinary decision was taken that he should be treated with a left hemicolectomy.

**Figure 1:**
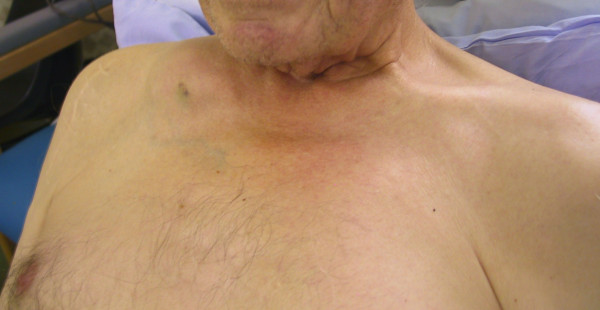
Clinical photograph of swelling in right supraclavicular fossa clearly demonstrating a swelling immediately superior to the medial aspect of the right clavicle.

**Figure 2:**
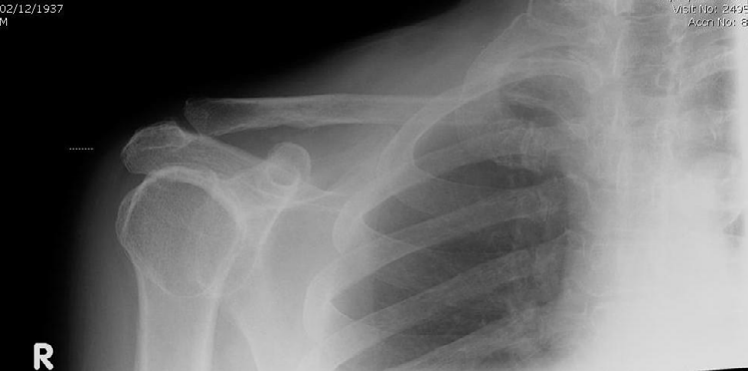
Plain radiograph of right clavicle. The radiograph demonstrates a pathological fracture of the medial aspect of the right clavicle.

Prior to the operation, he noticed a swelling in the right supraclavicular fossa (figure 1). The swelling was approximately 5 cm in diameter, with a hard consistency, immobile, non-tender, non-fluctuant and clinically appeared to be arising from the clavicle. There was no history of trauma. A plain X-ray showed an osteolytic lesion in the medial aspect of the right clavicle (figure 2).

**Figure 3:**
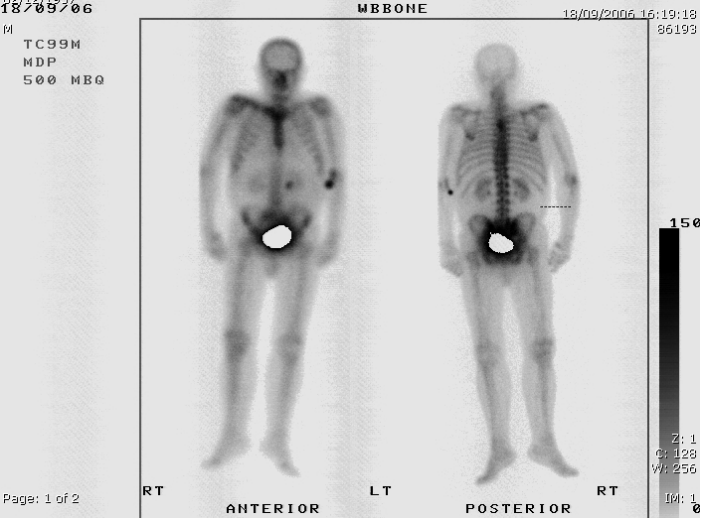
Isotope bone scan. The isotope bone scan shows no evidence of skeletal metastasis.

**Figure 4:**
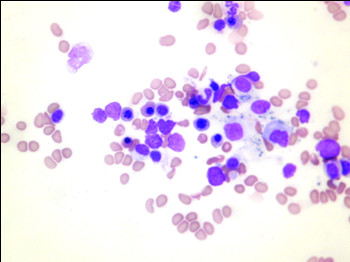
Cytology image of clavicular metastasis. This image reveals malignant cells arranged in cohesive clusters with nuclear pleomorphism, hyperchromasia and nuclear enlargement.

An isotope bone scan revealed diffuse uptake in the cervical vertebrae suggestive of degenerative changes but showed no evidence of metastases in the clavicle (figure 3). Hence, fine needle aspiration of the lesion was performed which revealed malignant cells arranged in cohesive clusters with nuclear pleomorphism, hyperchromasia and nuclear enlargement (figure 4). The malignant cells seen were compatible with metastases. At the same time, other causes of bony metastases were excluded using protein electrophoresis and computed tomography.

**Figure 5:**
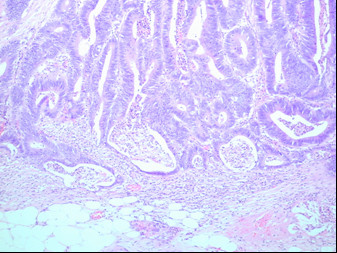
Histopathological image of resected bowel specimen. This images reveals an infiltrating adenocarcinoma with focal mucin pooling.

The patient underwent a palliative left hemicolectomy due to impending obstruction. Histology of the resected specimen revealed the following: macroscopic examination reveals a central annular cancer of 5 cms with perforation and mesenteric extension together with metastatic confluent marginal nodes (figure 5). Resection margins are clear. Microscopic examination confirms a hemicolectomy specimen with a large extensively ulcerated, focally perforated, and extensively infiltrating adenocarcinoma, with focal mucin pooling. Tumour perforation as stated has occurred. No evidence of any lymphovascular extension is seen. No evidence of lymphovascular involvement within the tumour bed, but 5 marginal nodes are positive out of a total of 12. Resection edges are free of tumour.

The patient had a stable postoperative course and was subsequently discharged home. Out-patient follow-up with the palliative care physicians was arranged and he received palliative radiotherapy to his clavicle and spine. Unfortunately, he died 2 months later.

## Discussion

Colorectal cancer is the third leading cause of cancer-related deaths in the world [2]. Colorectal cancer metastases are mainly seen in the liver (50%), lung (16%), skin (8%) and brain (8%). The incidence of skeletal metastases varies from 4 – 6% [3] and commonly arise in the pelvis or vertebral bones and rarely as an isolated bony metastatic lesion.

A detailed literature review showed that the clavicle is a very unusual site for metastasis from colorectal cancer and so far has been reported only once [1]. Bony metastases from primary colonic tumours do not have a clear pattern. They are mostly blood borne, probably through veins and they metastasise via the vertebral venous plexus to the vertebrae, pelvic bones, sacrum, skull, femur and humerus [3]. Occasionally, rare metastases to metacarpals, patella, sternum and mandible have been reported [4-6].

Osseous metastases are rarely a primary manifestation of bowel cancer [7] and are usually associated with other systemic manifestations in the liver, lung or brain [3]. The median time to bony metastasis varies from 7 to 13 months [7] with most of these arising from highly advanced rectal or sigmoid cancers [3]. One of the reasons for the increase in bony metastases at unusual sites could be improvements in adjuvant treatments resulting in improved survival, thus allowing time for the manifestation of atypical metastases[8].

Due to the rarity of skeletal metastases from colorectal cancer radiological investigations are undertaken only on clinical suspicion [3]; in our case the swelling was over the medial end of the clavicle. Most bony metastases are osteolytic and are described as a lesion greater than 1 cm in diameter with loss of 50% of the bone density, but occasionally they are osteoblastic [3]. Isotope bone scanning using Technetium 99 m phosphate compounds is probably used as the principal tool for diagnosing bone metastases. Although, it is more sensitive as compared to radiography, it fails to detect metastases on certain occasions [9]. Plausible explanations for false negative findings are pure osteolytic lesions growing rapidly, when bone turnover is slow, or when the site is avascular. Hence in case of equivocal findings alternative methods in terms of traditional needle biopsy [9] or more recently magnetic resonance imaging [10] can be used for diagnosis. Sometimes abnormal biochemistry can indicate bony metastases e.g. raised calcium or/and Alkaline phosphatase.

## Conclusion

This case report highlights that a high degree of suspicion should be employed in colorectal cancer patients presenting with bone pain or lesions. There is a need for caution when using isotope bone scanning for detecting bone metastases. With improved colorectal cancer survival and improved quality of care, it may be necessary to consider using bone scanning or MRI to identify and treat these lesions early.

### Learning Points

1. Solitary skeletal metastases from primary colorectal tumours are rare.

2. Skeletal metastases from primary colorectal tumours tend to occur in small, non-weight bearing bones including the clavicle.

3. Isotope bone scans should be used with caution in the detection of skeletal metastases. MRI or bone biopsy may be more useful diagnostic tools.

## Competing interests

The author(s) declare that they have no competing interests.

## Authors' contributions

NNBP – conceived the case report and drafted the manuscript

PRS – helped draft the manuscript

EW – obtained the images

PNH – edited the manuscript

All authors have read and approved the final manuscript.
